# Estimation of Bioactive Compound, Maslinic Acid by HPTLC, and Evaluation of Hepatoprotective Activity on Fruit Pulp of* Ziziphus jujuba* Mill. Cultivars in India

**DOI:** 10.1155/2016/4758734

**Published:** 2016-01-21

**Authors:** Anagha Rajopadhye, Anuradha S. Upadhye

**Affiliations:** Biodiversity and Palaeobiology, Agharkar Research Institute, G.G. Agarkar Road, Pune 411 004, India

## Abstract

Fruits of* Ziziphus jujuba* Mill. (family: Rhamnaceae), known as Indian jujube or “Ber,” are of potential nutritional and medicinal value. The objectives of the present study were to estimate bioactive compound maslinic acid by HPTLC method and to evaluate* in vitro* antioxidant and hepatoprotective activity of eight cultivars of Indian jujube. Maslinic acid and the fruit pulp of various cultivars of Indian jujube, namely,* Gola*,* Sannur*,* Umaran*,* Mehrun*, and* Chhuhara*, exhibited significantly high antioxidant and hepatoprotective activity. HPTLC-densitometric method was developed for quantification of maslinic acid from fruits of Indian jujube cultivars. The trend of occurrence of maslinic acid in fruits pulp extracts was as follows:* Gola* >* Sannur* >* Umaran* >* Mehrun* >* Chhuhara* > Wild >* Kadaka* >* Apple*. A significant correlation was shown by maslinic acid content and prevention of oxidative stress induced by CCl_4_ in liver slice culture cell treated with extract. Maslinic acid along with its other phytoconstituents like phenols, flavonoids, and ascorbic acid may act as a possible therapeutic agent for preventing hepatotoxicity caused by oxidative stress generated due to the prooxidants like CCl_4_. This is the first report of fruit pulp extracts of* Z. jujube* cultivars in India and maslinic acid preventing CCl_4_ induced damage in liver slice culture cell of mice.

## 1. Introduction

*Ziziphus jujuba* Mill. [syn. =* Ziziphus mauritiana* Lam.; family: Rhamnaceae], commonly known as Indian jujube or “Ber,” is an indigenous fruit crop of India. It is widely distributed throughout Old World tropics from Southern Africa through Middle East to the Indian Subcontinent. Fruits are eaten fresh or used in food products as dried, candied, squash, chutneys, pickles, and so forth for their potential nutritional and medicinal value [1]. It is also commonly used in the traditional system of medicines for curing various diseases such as digestive disorders, weakness, liver complaints, obesity, urinary troubles, diabetes, skin infections, loss of appetite, fever, bronchitis, anemia, and diarrhoea [2]. Chemical investigations of the fruit reveal the presence of a wide array of phytochemicals such as amino acids, ascorbic acid, flavonoids, phenolic acids, vitamins A and C, phosphorus, calcium, and iron [3–5]. In view of its phytoconstituents, most of the research on fruit deals with assessment of total antioxidant activity [6–11]. Fruits of jujube are reported for wide array of pharmacological activities such as antispastic, antifertility/contraception, hypotensive, antinephritic, cardiotonic, immunostimulant, anticancer, antibacterial, and antidiarrhoeal [12]. However, most of the information reported in the literature is restricted to Chinese jujube. Indian genotypes are still under research for their nutritional and therapeutic uses.

Fruits are the foremost source of a pentacyclic triterpene, maslinic acid [13] having diverse biological and pharmacological properties, such as antitumoural, cardiovascular, hepatoprotective, anti-inflammatory, antioxidant protection, antimicrobial, and anticarcinogenic activity [14].

A perusal of the literature shows hepatoprotective effect of* Z. jujube* [15, 16]. However, hepatoprotective activity of fruit pulp extract of different cultivars in India with respect to active component is not widely studied. The objectives of the present study were evaluation of* in vitro* hepatoprotective activity using liver slice culture model and estimation of maslinic acid in fruit pulp of* Z. jujuba* cultivars from India by High Performance Thin-Layer Chromatography (HPTLC).

## 2. Materials and Methods

### 2.1. Chemicals

2,2-Diphenyl-1-picrylhydrazyl (DPPH), 2,2-azinobis(3-ethylbenzothiazoline-6-sulphonic acid) (ABTS), potassium persulphate, butylated hydroxyl toluene (BHT), and maslinic acid were obtained from Sigma Aldrich, Fluka, USA. Ethylenediaminetetraacetic acid (EDTA), sodium chloride (NaCl), potassium chloride (KCl), calcium chloride (CaCl_2_), monopotassium phosphate (KH_2_PO_4_), magnesium sulphate (MgSO_4_), carbon tetrachloride (CCl_4_), ascorbic acid, hydrochloric acid, potassium phosphate, glucose, Folin-Ciocalteu, other analytical grade solvents, and regents were purchased from S.D. Fine Chemicals, Mumbai, India.

### 2.2. Plant Material

Fruits of* Umaran*,* Chhuhara*, and* Gola*, three different cultivars* Z. jujuba*, were collected from Solapur (Maharashtra, India) during postmonsoon season of 2011-2012. Fruits of* Mehrun* and wild variety were collected from Dhule (Maharashtra, India) and campus of Agharkar Research Institute, Pune, during the same season. Fruits of* Sannur*,* Kadaka*, and* Apple* were purchased from Pune (Maharashtra, India) local markets. Plant samples were authenticated by the Biodiversity and Palaeobiology Group, Agharkar Research Institute, Pune 411 004. Fruits were washed with tap water and pulp was separated from the seeds. Edible part of the fruits was cut into small pieces and freeze dried in a lyophilizer (Thermo Savant ModulyoD, USA). All samples were ground into powder and stored in freezer until analyzed.

### 2.3. Preparation of Sample Extract

Powdered samples (25 g) were extracted with methanol placed in the stainless-steel cell of a Dionex ASE^TM^ 100 system (Sunnyvale, CA, USA). Extraction was performed at 100 bar and at three temperatures, 60°C, for 15 min with five replicate cycles. Extracts were concentrated under reduced temperature and pressure using rotary evaporator. Respective yields of methanol extracts were as follows:* Umaran*, 2.015 g,* Chhuhara*, 1.503 g,* Mehrun*, 2.57 g,* Gola*, 1.490 g,* Sannur*, 1.677 g,* Kadaka*, 1.390 g,* Apple*, 2.305 g, and Wild, 1.03 g.

### 2.4. Antioxidant Activity

Abilities of the samples in scavenging DPPH radical were determined as per the standard method [17]. Further, *β*-carotene linoleic acid and ABTS assays were performed according to the reported method [18, 19]. Sample concentration providing 50% inhibition (IC_50_) was calculated and reported as mean ± SD. BHT was used as reference compound.

The method of photochemiluminescence (PCL) was used for determination of integral antioxidant capacity (IAC) of lipid soluble antioxidant substances. The equipment used was Photochem^®^ with standard kit antioxidant capacity of water-soluble compounds (AWL) and lipid soluble compound (ACL) (Analytik Jena AG). Calibration and measurement were performed according to standard kit protocol.

### 2.5. *In Vitro* Hepatoprotective Activity

#### 2.5.1. Animals

To assess hepatoprotective activity, adult albino mice (6–8 weeks old) of either sex breed in animal house of Agharkar Research Institute, Pune, were used for preparation of liver slices. Approval for this work, using animals, was taken from the Institutional Animal Ethical Committee (number 101/1999/CPCSEA, August 1, 2011) of Agharkar Research Institute, Pune.

#### 2.5.2. Liver Slice Culture

Liver slice culture was maintained following the protocol developed by Wormser and Ben-Zakine [20] and Invittox protocol number 42 [21]. Mice were dissected open after cervical dislocation, and liver lobes were removed and transferred to prewarmed Kred's Ringer Hepes (KRH) (2.5 mM Hepes, pH 7.4, 11 8 mM NaCl, 2.85 mM KCl, 2.5 mM CaCl_2_, 1.5 mM KH_2_PO_4_, 1.18 mM MgSO_4_, 5 mM *β*-hydroxybutyrate, and 4.0 mM glucose). Liver was cut into thin slices using sharp blade. Slices were weighed and those weighing between 4 and 6 mg were used for the experiment. Each experimental system contained 20–22 slices weighing together 100–120 mg. These slices were washed with 10 mL KRH medium, every 10 min over a period of 1 h. These were then preincubated for 60 min in small plugged beakers containing 2 mL KRH on a shaker water bath at 37°C. At the end of preincubation, the medium was replaced by 2 mL of fresh KRH and incubated for 2 h at 37°C.

#### 2.5.3. Experiment Design

Liver slices were further divided into individual cultures for further respective treatments: Set 1: control; Set 2: 15.5 mM CCl_4_; Set 3: 100 *μ*g/mL sample extracts; Set 4: 10 *μ*g/mL maslinic acid; Set 5: 15.5 mM CCl_4_ + 100 *μ*g/mL sample extracts; Set 6: 15.5 mM CCl_4_ + 10 *μ*g/mL maslinic acid; Set 7: 15.5 mM CCl_4_ + 10 mM ascorbic acid; Set 8: 10 mM ascorbic acid.

After respective treatments, all the cultures were incubated in constant temperature water bath at 37°C for 2 h. At the end of incubation, each group of slices was homogenized in appropriate volume of chilled potassium phosphate buffer (100 mM, pH 7.8) in an ice bath to give a tissue concentration of 100 mg/mL. Culture medium was collected and used for estimation of lactate dehydrogenase (LDH), which was employed as a cytotoxicity marker. Homogenates were centrifuged at 10,000 rpm for 10 min at 4°C and supernatants assayed for LDH, catalase, peroxidase, and superoxide dismutase. All these were measured following the instructions on commercial kits (Accurex Biomedical Pvt. Ltd., India). Lipid peroxidation was determined in terms of malondialdehyde (MDA). Ascorbic acid (AA) was used as standard.

#### 2.5.4. Data Analysis

Data were expressed as mean ± standard deviation for three parallel experiments. The results of treatment effects were analyzed using one-way ANOVA test (Graphpad Prism 4) and *p* values < 0.001 were considered as highly significant and *p* values < 0.05 were considered as significant.

### 2.6. HPTLC Analysis of Maslinic Acid

#### 2.6.1. Chromatographic Conditions

HPTLC was performed on 20 × 10 cm normal phase HPTLC aluminum plates (Merck, Darmstadt, Germany) precoated with 200 mm layer thickness of silica gel 60 F_254_. The samples were applied at 10 mm from the lower edge of the plates, 10 mm apart, and maximum fourteen samples with bandwidth 6 mm using Linomat IV sample applicator (Camag, Switzerland) fitted with a microliter syringe. Chromatography was performed in Automatic Developing Chamber (Camag, ADC 2) previously saturated with mobile phase vapors for 15 min at 28 ± 2°C with toluene : ethyl acetate (6.5 : 3.5 v/v) as mobile phase. TLC plates were dipped in freshly prepared anisaldehyde sulphuric acid reagent and heated at 105°C for 5 min. Densitometry scanning was performed at *λ* 592 nm using a Camag TLC scanner 3 with winCATS software.

#### 2.6.2. Preparation of Standard Solution and Calibration Plot for Maslinic Acid

Standard solutions of maslinic acid in the range 150–750 ng/*μ*L were prepared by diluting the stock solution of 1 mg/mL. Respective concentration of standard solution (10 *μ*L) was applied on the plate with bandwidth 6 mm and developed under optimized conditions. Procedure was repeated three times to plot a graph of response (peak area) and amount of maslinic acid.

#### 2.6.3. Estimation of Maslinic Acid in Methanol Extracts of Fruit Samples

Suitable quantities of extracts (5 *μ*L) from eight cultivars were applied in replicates on silica gel F_254_ HPTLC plates. Plates were developed and scanned using standardized conditions and peak areas were recorded. A calibration plot was obtained by plotting peak area against quantity of maslinic acid standard, and the amounts of maslinic present in the samples were calculated using the calibration plot.

#### 2.6.4. Validation of Method

Method was validated according to ICH guidelines by determining specificity, limit of detection (LOD), and limit of quantification (LOQ). Precision of the instrumentation was checked by replicate (*n* = 6) scanning of same bands of maslinic acid (150 ng per band) and calculation of RSD [%]. Repeatability of the method was tested by replicate (*n* = 6) scanning of bands obtained from standard solutions of maslinic acid (150 ng per band) after application to a HPTLC plate. Variability of the method was studied by analysis of bands obtained from standard solutions of maslinic acid of different concentration (150, 450, and 750 ng per band) on same day (intraday precision) and on different days (interday precision), and RSD [%] was calculated. Robustness of the method was studied at two different concentrations of maslinic acid, 150, and 300 ng per band by introducing small deliberate changes in mobile phase composition toluene : ethyl acetate (6.3 : 3.7 and 6.7 : 3.3 v/v) in triplicate. Accuracy of the method was tested by determination of recovery at three levels. Preanalyzed samples were spiked with extra maslinic acid (50, 100, and 150%) and the samples were reanalyzed.

Percentage recovery and average percentage recovery were calculated. Limits of detection (LOD) and quantification (LOQ) were calculated by use of the equations; LOD = 3.3 × SD/*S* and LOQ = 10 × SD/*S*, respectively, where SD is the standard deviation and *S* is the slope of the calibration plot, with defined precision and accuracy under the given experimental conditions.

## 3. Results

### 3.1. Antioxidant Activity

**Figure 1:**
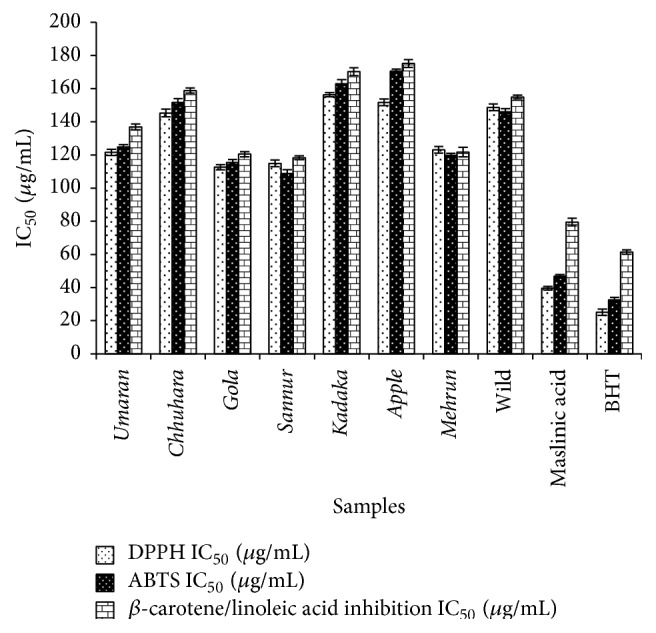
Comparative antioxidant activity of fruit pulp extracts of eight cultivars of* Z. jujuba* and maslinic acid. DPPH: 2,2-diphenyl-1-picrylhydrazyl; ABTS: 2,2-azinobis(3-ethylbenzothiazoline-6-sulphonic acid). Values are mean ± SD of three experiments.

The fleshy mesocarp (pulp) of different cultivars of jujube fruit is consumed all over India. Hence, in the present study, fruit pulp of different cultivars was used to investigate antioxidant potential in terms of scavenging of DPPH, ABTS, and *β*-carotene/linoleic acid photochemiluminescence* in vitro* systems (Figure 1 and Table 1).

DPPH and ABTS are commercially available stable free radicals commonly used as substrate, to evaluate antioxidant capacity. In DPPH assay, antioxidants from the sample scavenge free radical by hydrogen donation which causes a reduction in the intensity of absorption at 517 nm. In contrast to DPPH, the sample to be tested was added after generation of a certain amount of ABTS^•+^ radical cation and the remaining ABTS^•+^ concentration after reaction with antioxidant sample was then quantified. In the present study, antioxidant activity was found to vary for different cultivars of jujube fruits. IC_50_ value for maslinic acid (39.6 ± 1.12 *μ*g/mL) is comparable with standard BHT (28.12 ± 1.98 *μ*g/mL). Antioxidant activities (IC_50_ values) of the fruit pulp of jujube were in the range of 112.6 ± 1.89 to 156.3 ± 1.24 *μ*g/mL and 115.4 ± 1.89 to 170.5 ± 1.29 *μ*g/mL as determined by the DPPH and ABTS assays, respectively. The order of hierarchy was as follows: maslinic acid >* Gola* >* Sannur* >* Umaran* >* Mehrun* >* Chhuhara* > Wild >* Kadaka* >* Apple* (Figure 1).

The capacity of the different cultivars of jujube extracts to inhibit lipid peroxidation was evaluated using *β*-carotene/linoleic acid bleaching method. The oxidation of linoleic acid generates peroxyl free radicals which then oxidize the highly unsaturated *β*-carotene. The oxidation of *β*-carotene decreases by the antioxidants present in the sample. Maslinic acid from the fruit pulp of the eight cultivars, namely,* Gola*,* Sannur*,* Umaran*,* Mehrun*, and* Chhuhara*, showed high capacity towards the inhibition of *β*-carotene bleaching with low IC_50_ value (39.6 ± 1.12, 112.6 ± 1.56, 114.9 ± 2.01, 121.5 ± 1.89, 123.1 ± 2.03, and 145.3 ± 2.34 *μ*g/mL) comparable with standard BHT (25.1 ± 1.90 *μ*g/mL).

The Photochem method is easy and rapid to perform for evaluation of antioxidant capacity in which superoxide radical (O_2_^∙−^) is generated at room temperature. The PCL assay is conducted to determine integral antioxidant capacity (IAC) which represents the antioxidant capacity of hydrophilic (ACW) and lipophilic antioxidants (ACL), calculated as nmol equivalents in activity of Trolox/ascorbic acid [22]. Luminol works as both photosensitizer and O_2_^∙−^ detection reagent.

In this assay, the radical scavenging property of antioxidant is determined against O_2_^∙−^ which is a final venomous byproduct of oxygen metabolism responsible for the most important damage related to reperfusion injuries. Fruit pulp extracts of eight cultivars of jujube were found to be distinctly varied in ACW and ACL values. ACL values for these cultivars of jujube and maslinic acid were found to be higher than ACW. The order of hierarchy for IAC was as follows: maslinic acid >* Gola* >* Sannur* >* Umaran* >* Mehrun* >* Chhuhara* > Wild >* Kadaka* >* Apple* (Table 1).

### 3.2. *In Vitro* Hepatoprotective Activity

The liver slice culture is an* in vitro* technique consisting of a highly organized cellular community in which different cell types are subjected to mutual contact. It provides desirable complexity of structurally and functionally intact cells which are valuable approaches for screening of plant extracts/fractions/pure compounds for their hepatoprotective activity and elucidation of the possible mechanism of actions [23, 24].

Cytotoxic CCl_4_ was used to induce oxidative stress to the liver slice culture. Release of LDH in the liver slice culture medium was used as cytotoxicity marker. CCl_4_ was highly toxic to the treated cells as concentration of LDH was increased in the medium as compared to control. Fruit pulp extracts of eight cultivars of jujube were found to be nontoxic at dose of 100 *μ*g/mL as it showed percentage release of LDH in the medium similar to that of control untreated slices.

LDH release in the culture system treated with CCl_4_ was found to be 5.55 times more as compared to control. After addition of samples extract along with CCl_4_ cytotoxicity, the amount of LDH release in medium reduced significantly (*p* < 0.001, *p* < 0.05) (Table 2). Maslinic acid and fruit pulp extracts of* Gola*,* Sannur*,* Umaran*,* Mehrun*, and* Chhuhara* showed very significant (*p* < 0.001) reduction of LDH release in the medium.

Time course of lipid peroxidation and LDH release were assessed in the presence of cytotoxic agent alone and together with different extracts and maslinic acid. Continuous LDH release was observed up to 2 h by the liver tissue treated with CCl_4_ only, whereas the LDH release by the control (untreated) was constant. However, in presence of maslinic acid and fruit pulp extracts of* Gola*,* Sannur*,* Umaran*,* Mehrun*, and* Chhuhara* along with cytotoxic agent treated liver tissue showed increase in the LDH up to 1 h incubation. Further, significant decrease in LDH activity (*p* < 0.001) was comparable with standard ascorbic acid (Table 3).

CCl_4_ is known to generate oxidative stress in cells, which can be measured from the extent of lipid peroxidation in liver tissue. The lipid peroxidation levels in the liver slice culture medium were assessed by TBARS assay. Lipid peroxidation was measured in terms of thiobarbituric acid reactive substances and was expressed as *μ*mol of malondialdehyde formed/100 mg tissue. The continuous increase of MDA (2 fold) was observed up to 2 h incubation in the liver slice treated with CCl_4_ alone, compared to control. The reduction of lipid peroxidation was found to be significantly (*p* < 0.001) near control levels in liver cells treated with either maslinic acid or fruits pulp extracts of* Gola*,* Sannur*,* Umaran*,* Mehrun*, and* Chhuhara* along with CCl_4_ cytotoxicity (Table 3).

CCl_4_ induces oxidative stress in the cells by generation of ROS. Highly reactive free radicals generated from the oxidative stress protect by antioxidant enzymes (AOEs) SOD, CAT, and GR. SOD and CAT are known enzymes to prevent damage by directly scavenging the harmful active oxygen species. GR plays a role in recycling the oxidized glutathione to reduced glutathione, which acts as an antioxidant [24]. Activities of AOEs were assessed in liver slice cultures treated with CCl_4_ alone or CCl_4_ and extracts/maslinic acid. The liver tissue treated with either maslinic acid or fruits pulp extracts of* Gola*,* Sannur*,* Umaran*,* Mehrun*, and* Chhuhara* along with CCl_4_ cytotoxicity showed significant (*p* < 0.001) reduction of antioxidant enzymes (Table 2). The activity of maslinic acid or fruit pulp extracts of* Gola*,* Sannur*,* Umaran*,* Mehrun*, and* Chhuhara* was comparable with standard.

### 3.3. HPTLC Analysis of Maslinic Acid in Fruit Pulp Extracts of Different Jujube Cultivars

The maslinic acid showed significantly potential antioxidant and hepatoprotective activities* in vitro*; hence, quantification of this bioactive molecule was carried out by HPTLC method.

**Figure 2:**
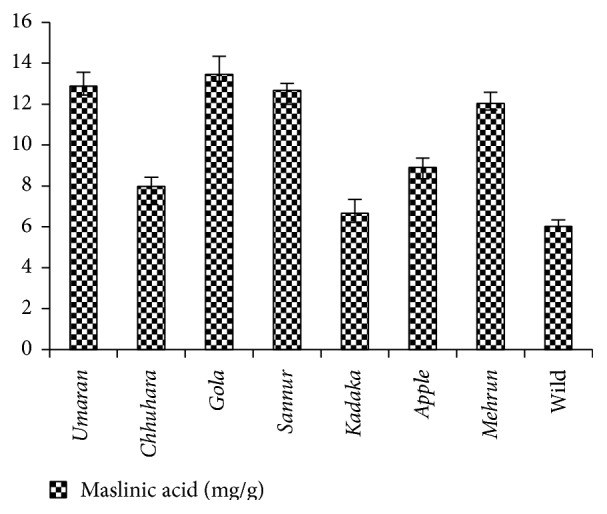
Comparative maslinic acid contents in fruit pulp of eight cultivars of* Z. jujuba*.

Chromatographic separation was achieved in system containing toluene : ethyl acetate (6.5 : 3.5 v/v). It gave optimized result with sharp, symmetrical, and well-resolved peak of maslinic acid at R_F_ 0.21 from other components of the samples extract. A linear relationship was obtained between response (peak area) and amount of maslinic acid in the range of 150–750 ng per band; the correlation coefficient was *r* = 0.9903 (*Y* = 29.616 + 0.343*∗X*) (see Table S1 and Figures S1A and S1B in Supplementary Material available online at http://dx.doi.org/10.1155/2016/4758734). Maslinic acid was observed in chromatographs of all cultivars of fruit pulp samples extract with variation in their contents. It was higher in fruit pulp extracts of* Gola*,* Sannur*,* Umaran*,* Mehrun*, and* Chhuhara*. The trend of occurrence of maslinic acid in various fruit pulp extracts was as follows:* Gola* >* Sannur* >* Umaran* >* Mehrun* >* Chhuhara* > Wild >* Kadaka* >* Apple* (Figure 2).

The method was validated for precision, repeatability, and accuracy (Tables S1–S3). The repeatability (intraday precision) and intermediate (interday) precision were calculated (expressed as % RSD) as of data obtained during six-day validation which was observed in the range of 1.15 to 3.41 and 2.87 to 4.09, respectively (Table S2). Instrument precision was checked by repeated scanning of maslinic acid band at 150 ng (*n* = 6) and expressed as relative standard deviation (% RSD = 0.67) (Table S1). Small changes in mobile phase composition had no significant effect on the chromatographic profile. The accuracy of the method was tested by determination of recovery at three levels. The changes in mobile phase composition did not show considerable effect on resolution and R_F_ of compound. The low RSD values of the peak areas calculated indicate the robustness of the method (0.89). Recovery varied between 93.22 and 97.41% (Table S3). High recovery indicated that the proposed method was reliable and reproducible. The LOD and LOQ were 30 and 87 ng per spot, respectively (Table S1). The purity of the maslinic acid peak was checked from the samples by recording UV spectra. The identified maslinic spot was confirmed from sample extracts by overlaying UV absorption spectrum of samples with standard at 592 nm (Figure S1C).

## 4. Discussion

Many fruits grown in a tropical and subtropical climate have been evaluated for their nutritional value and health benefits. It contains high concentrations of nutrients and natural phytochemicals having diverse pharmacological actions such as anti-inflammatory, anticancer, antidiabetic, hepatoprotective, and antioxidant activities. In view of the growing interest in these compounds, it is necessary to identify and quantify these valued compounds in fruits for the therapeutic uses [25]. In the present study,* in vitro* antioxidant and hepatoprotective activity of the fruits pulp of eight commonly used cultivars of jujube were evaluated. Further, bioactive compound, maslinic acid, was quantified by HPTLC method for correlation of content of maslinic acid and bioactivity of fruit pulp of eight cultivars of jujube.

In addition, evaluation of the antioxidant activity is becoming increasingly relevant in the field of nutrition as it provides useful information with regard to health promoting and functional quality of the raw material. In the present study, antioxidant potential was investigated for fruit pulp of eight cultivars of jujube. Hitherto, most of the reports on jujube deal with the evaluation of total antioxidant activity of extracts with respect to their phenolic, flavonoids, and ascorbic acid contents [6–11]. However, no information could be available for other types of compounds contained in jujube fruit, such as maslinic acid, a triterpenic bioactive constituent. In the present study, maslinic acid and, out of the eight cultivars tested, fruits pulp extracts of* Gola*,* Sannur*,* Umaran*,* Mehrun*, and* Chhuhara* exhibited antioxidant potential which supported previous reports [11]. A similar observation was found for the hepatoprotective activity. A simple, sensitive, and cost effective HPTLC-densitometric method has been developed for detection and quantification of maslinic acid in fruits of Indian jujube cultivars. Maslinic acid content was higher in* Gola*,* Sannur*,* Umaran*,* Mehrun*, and* Chhuhara*. Maslinic acid contents in eight cultivars of jujube and* in vitro* hepatoprotective activity were associated significantly.

## 5. Conclusion

We report for the first time* in vitro* antioxidant and hepatoprotective activity of maslinic acid and fruit pulp of eight cultivars of* Ziziphus jujuba*. Results showed that the pretreatment with fruit pulp extracts was effective against CCl_4_ induced toxicity in the liver. Maslinic acid along with its other phytoconstituents like phenolic acid, flavonoids, and ascorbic acid may act as a possible therapeutic agent for preventing hepatotoxicity caused by the oxidative stress generated due to the prooxidants like CCl_4_. This study suggests that fruit pulp of jujube can play a vital role in prevention of liver damage caused by chemicals and it can be used as a potent hepatoprotective agent. Further, detailed investigations on identification of nutritionally superior cultivars of* Z. jujuba* could potentially interact with other pharmaceuticals. These cultivars may be highly desirable in germplasm breeding programs to breed quality varieties of* Z. jujuba* with high antioxidant potential.

## Supplementary Material

HPTLC method was validated in terms of peak purity, precision, LOD, LOQ and accuracy according to ICH guidelines (2005). The method was specific for analysis of active principle maslinic acid in fruit pulp samples extract of eight cultivars of *Z. jujuba*.

## Figures and Tables

**Table 1:** Integral antioxidant capacity (IAC) of fruit pulp extracts of eight cultivars of *Z. jujuba* and maslinic acid: water-soluble antioxidant capacity, corresponding to the activity expressed as nmol equivalents of ascorbic acid for each gram of tested extract; lipid soluble antioxidant capacity, corresponding to the activity expressed as nmol equivalents of Trolox for each gram of tested extract.

Sample	Photochemiluminescence-ascorbic acid equivalent (nmol/g)	Photochemiluminescence ACL in Trolox equivalent (nmol/g)
*Umaran*	40.67 ± 2.67	27.89 ± 1.98
*Chhuhara*	42.67 ± 2.43	34.56 ± 1.05
*Gola*	38.99 ± 2.12	28.56 ± 1.12
*Sannur*	41.23 ± 2.78	27.90 ± 1.23
*Kadaka*	56.78 ± 2.89	57.98 ± 1.56
*Apple*	60.78 ± 1.98	45.89 ± 1.11
*Mehrun*	38.92 ± 2.33	29.65 ± 0.98
Wild	59.87 ± 1.87	55.78 ± 0.67
Maslinic acid	34.56 ± 2.34	10.89 ± 0.91

Values are mean ± SD of three experiments.

**Table 2:** Effect of fruit pulp extracts of eight cultivars of *Z. jujuba *and maslinic acid in protecting liver cells from CCl_4_ induced cytotoxicity by ameliorating oxidative stress.

Treatment	LDH units/100 mg tissue wet wt.	SOD units/100 mg tissue wet wt.	CAT units/100 mg tissue wet wt.	GR units/100 mg tissue wet wt.
Control	7.83 ± 1.16	17.83 ± 1.47	15.00 ± 1.41	0.151 ± 0.012
CCl_4_	43.53 ± 1.63	56.33 ± 2.25	87.5 ± 1.04	0.498 ± 0.008
AA	7.53 ± 1.47	17.5 ± 2.14	15.33 ± 1.75	0.150 ± 0.007
100 *μ*g/mL UM	7.00 ± 2.09	17.43 ± 1.72	15.65 ± 1.37	0.156 ± 0.003
100 *μ*g/mL CH	8.33 ± 1.16	17.23 ± 1.47	15.83 ± 1.22	0.159 ± 0.011
100 *μ*g/mL GO	7.17 ± 1.47	17.0 ± 1.41	15.67 ± 1.67	0.158 ± 0.006
100 *μ*g/mL SA	7.49 ± 1.13	17.60 ± 1.56	15.66 ± 1.45	0.153 ± 0.012
100 *μ*g/mL KA	7.13 ± 0.98	17.89 ± 1.23	15.78 ± 1.43	0.155 ± 0.009
100 *μ*g/mL AP	7.91 ± 1.23	17.56 ± 1.45	15.06 ± 1.72	0.153 ± 0.006
100 *μ*g/mL ME	7.55 ± 1.56	17.33 ± 1.76	15.78 ± 1.76	0.158 ± 0.002
100 *μ*g/mL WI	7.34 ± 1.89	17.09 ± 1.45	15.03 ± 1.29	0.156 ± 0.005
10 *μ*g/mL MA	8.01 ± 1.76	17.21 ± 1.34	15.34 ± 1.09	0.154 ± 0.006
CCl_4_ + 100 *μ*g/mL UM	19.83 ± 1.21^*∗*^	22.17 ± 1.16^*∗*^	25.74 ± 2.28^*∗*^	0.206 ± 0.008^*∗*^
CCl_4_ + 100 *μ*g/mL CH	22.74 ± 2.48^*∗*^	24.5 ± 0.54^*∗*^	28.24 ± 1.09^*∗*^	0.236 ± 0.008^*∗*^
CCl_4_ + 100 *μ*g/mL GO	19.33 ± 1.98^*∗*^	20.78 ± 0.67	22.77 ± 2.89	0.213 ± 0.004
CCl_4_ + 100 *μ*g/mL SA	27.0 ± 0.89^*∗*^	38.17 ± 1.94^*∗*^	36.67 ± 0.81^*∗*^	0.298 ± 0.008^*∗*^
CCl_4_ + 100 *μ*g/mL KA	34.33 ± 1.04^a^	32.0 ± 0.89^a^	44.67 ± 1.03^a^	0.362 ± 0.008^a^
CCl_4_ + 100 *μ*g/mL AP	39.33 ± 0.81^a^	48.67 ± 0.81^a^	47.83 ± 0.75^a^	0.386 ± 0.005^a^
CCl_4_ + 100 *μ*g/mL ME	21.33 ± 2.48^*∗*^	23.5 ± 0.57^*∗*^	27.44 ± 1.39^*∗*^	0.256 ± 0.008^*∗*^
CCl_4_ + 100 *μ*g/mL WI	39.33 ± 0.81^a^	48.67 ± 0.81^a^	47.83 ± 0.75^a^	0.386 ± 0.055^a^
CCl_4_ + 10 *μ*g/mL MA	15.83 ± 1.21^*∗*^	19.17 ± 1.16^*∗*^	23.74 ± 2.28^*∗*^	0.216 ± 0.003^*∗*^
CCl_4_ + 50 mM AA	13.67 ± 1.03^*∗*^	15.33 ± 1.63^*∗*^	19.83 ± 1.83^*∗*^	0.189 ± 0.018^*∗*^

Cytotoxicity was assessed in terms of % lactate dehydrogenase (LDH) released, and the response to oxidative stress was measured in terms of antioxidant enzymes. SOD: superoxide dismutase; CAT: catalase; GR: glutathione reductase activity. Ascorbic acid (AA) was used as a standard. UM:* Umaran*; CH:* Chhuhar*; GO:* Gola*; SA:* Sannur*; KA:* Kadaka*; AP:* Apple*; ME:* Mehrun*; WI: Wild; MA: maslinic acid.

Values represent means of at least three experiments and their standard deviation.

^a^Significantly different compared with respective CCl_4_ treated group, *p* < 0.05.

^*∗*^Significantly different compared with respective CCl_4_ treated group, *p* < 0.001.

**Table 3:** Percentage release of LDH and extent of lipid peroxidation in liver slice culture in CCl_4_ induced cytotoxicity.

	Treatment	Time at which activity was measured			
		0.5 h	1 h	1.5 h	2 h
% LDH release	Control	3.67 ± 0.97	4.33 ± 0.95	5.67 ± 1.12	7.82 ± 1.23
	CCl_4_	29.32 ± 1.34	34.55 ± 1.23	37.78 ± 1.56	43.98 ± 1.28
	AA	4.88 ± 0.98	5.09 ± 0.95	7.01 ± 1.16	7.34 ± 1.21
	100 *μ*g/mL UM	4.23 ± 0.87	5.13 ± 0.66	6.59 ± 1.23	7.67 ± 1.56
	100 *μ*g/mL CH	4.66 ± 0.97	4.91 ± 0.95	5.77 ± 1.12	7.55 ± 1.23
	100 *μ*g/mL GO	4.39 ± 1.34	5.60 ± 1.23	6.01 ± 1.56	6.93 ± 1.28
	100 *μ*g/mL SA	4.02 ± 1.56	5.12 ± 1.56	6.89 ± 1.12	7.08 ± 1.51
	100 *μ*g/mL KA	4.12 ± 1.34	5.04 ± 0.95	6.45 ± 0.98	7.56 ± 1.34
	100 *μ*g/mL AP	4.56 ± 1.12	5.45 ± 1.12	6.23 ± 1.58	8.01 ± 1.89
	100 *μ*g/mL ME	4.78 ± 1.78	6.01 ± 1.23	6.79 ± 1.23	7.34 ± 1.56
	100 *μ*g/mL WI	4.71 ± 1.23	5.98 ± 1.34	6.90 ± 1.77	8.12 ± 1.43
	10 *μ*g/mL MA	4.55 ± 1.66	5.78 ± 1.28	6.55 ± 1.56	7.22 ± 1.67
	CCl_4_ + 100 *μ*g/mL UM	23.45 ± 1.12^*∗*^	33.71 ± 1.34^*∗*^	25.13 ± 1.34^*∗*^	21.41 ± 1.89^*∗*^
	CCl_4_ + 100 *μ*g/mL CH	22.76 ± 1.67^*∗*^	32.17 ± 1.11^*∗*^	24.53 ± 1.44^*∗*^	21.45 ± 1.67^*∗*^
	CCl_4_ + 100 *μ*g/mL GO	21.64 ± 0.67^*∗*^	30.37 ± 1.79^*∗*^	24.09 ± 1.12^*∗*^	20.97 ± 1.56^*∗*^
	CCl_4_ + 100 *μ*g/mL SA	23.78 ± 1.12^*∗*^	34.88 ± 1.09^*∗*^	27.04 ± 1.15^*∗*^	23.88 ± 1.71^*∗*^
	CCl_4_ + 100 *μ*g/mL KA	27.89 ± 1.23^a^	33.56 ± 1.15^a^	27.81 ± 1.12^a^	24.78 ± 1.51^a^
	CCl_4_ + 100 *μ*g/mL AP	28.45 ± 0.96^a^	34.01 ± 1.34^a^	28.79 ± 1.15	26.33 ± 1.66
	CCl_4_ + 100 *μ*g/mL ME	23.05 ± 1.66^*∗*^	32.44 ± 1.02^*∗*^	25.67 ± 1.08^*∗*^	22.66 ± 1.14^*∗*^
	CCl_4_ + 100 *μ*g/mL WI	29.08 ± 0.98^a^	32.16 ± 0.95^a^	28.82 ± 1.12^a^	26.28 ± 1.2^a^
	CCl_4_ + 10 *μ*g/mL MA	20.34 ± 1.01^*∗*^	24.55 ± 1.67^*∗*^	19.67 ± 1.23^*∗*^	15.33 ± 1.77^*∗*^
	CCl_4_ + 50 mM AA	18.58 ± 0.97^*∗*^	21.69 ± 0.95^*∗*^	15.27 ± 1.12^*∗*^	13.27 ± 1.23^*∗*^

*μ*moles of MDA	Control	1.63 ± 0.14	2.18 ± 0.15	2.23 ± 0.08	2.29 ± 0.18
	CCl_4_	2.31 ± 0.17	2.89 ± 0.15	3.36 ± 0.15	4.02 ± 0.14
	AA	1.71 ± 0.18	2.02 ± 0.18	2.03 ± 0.16	2.16 ± 0.14
	100 *μ*g/mL UM	1.83 ± 0.28	2.01 ± 0.15	2.11 ± 0.17	2.18 ± 0.19
	100 *μ*g/mL CH	1.69 ± 0.14	2.09 ± 0.21	2.11 ± 0.19	2.16 ± 0.16
	100 *μ*g/mL GO	1.62 ± 0.13	2.01 ± 0.24	2.13 ± 0.22	2.21 ± 0.24
	100 *μ*g/mL SA	1.45 ± 0.15	2.11 ± 0.78	2.67 ± 0.34	2.72 ± 0.66
	100 *μ*g/mL KA	1.72 ± 0.45	2.01 ± 0.77	2.16 ± 0.12	2.23 ± 0.71
	100 *μ*g/mL AP	1.98 ± 0.79	2.05 ± 0.34	2.25 ± 0.56	2.67 ± 0.51
	100 *μ*g/mL ME	1.23 ± 0.12	1.67 ± 0.22	2.09 ± 0.14	2.27 ± 0.56
	100 *μ*g/mL WI	1.71 ± 0.48	1.98 ± 0.56	2.17 ± 0.23	2.87 ± 0.32
	10 *μ*g/mL MA	1.12 ± 0.44	1.98 ± 0.56	2.05 ± 0.31	2.45 ± 0.28
	CCl_4_ + 100 *μ*g/mL UM	2.04 ± 0.09^*∗*^	2.36 ± 0.11^*∗*^	2.85 ± 0.24^*∗*^	2.47 ± 0.28^*∗*^
	CCl_4_ + 100 *μ*g/mL CH	2.11 ± 0.15^*∗*^	2.43 ± 0.18^*∗*^	2.94 ± 0.09^*∗*^	2.31 ± 0.14^*∗*^
	CCl_4_ + 100 *μ*g/mL GO	2.10 ± 0.89^*∗*^	2.76 ± 0.15^*∗*^	2.91 ± 0.81^*∗*^	2.45 ± 0.66^*∗*^
	CCl_4_ + 100 *μ*g/mL SA	2.24 ± 0.51^*∗*^	2.57 ± 0.28^*∗*^	2.91 ± 0.93^*∗*^	2.45 ± 0.48^*∗*^
	CCl_4_ + 100 *μ*g/mL KA	2.51 ± 0.12^a^	3.22 ± 0.55^a^	3.49 ± 0.11^a^	3.02 ± 0.78^a^
	CCl_4_ + 100 *μ*g/mL AP	2.41 ± 0.12^a^	3.02 ± 0.55^a^	3.31 ± 0.11^a^	2.99 ± 0.78^a^
	CCl_4_ + 100 *μ*g/mL ME	2.26 ± 0.45^*∗*^	2.53 ± 0.81^*∗*^	2.97 ± 0.29^*∗*^	2.38 ± 0.19^*∗*^
	CCl_4_ + 100 *μ*g/mL WI	2.34 ± 0.22^a^	2.99 ± 0.25^a^	3.22 ± 0.14^a^	3.03 ± 0.18^a^
	CCl_4_ + 10 *μ*g/mL MA	1.82 ± 0.48^*∗*^	2.76 ± 0.31^*∗*^	2.81 ± 0.75^*∗*^	2.45 ± 0.56^*∗*^
	CCl_4_ + 50 mM AA	1.72 ± 0.21^*∗*^	2.21 ± 0.19^*∗*^	2.53 ± 0.20^*∗*^	2.25 ± 0.29^*∗*^

CCl_4_: carbon tetrachloride; AA: standard ascorbic acid, at 50 mM concentration; LPO: lipid peroxidation; LDH: lactate dehydrogenase; UM:* Umaran*; CH:* Chhuhar*; GO:* Gola*; SA:* Sannur*; KA:* Kadaka*; AP:* Apple*; ME:* Mehrun*; WI: Wild; MA: maslinic acid.

Values are mean of three experiments.

^a^Significantly different compared with respective CCl_4_ treated group, *p* < 0.05.

^*∗*^Significantly different compared with respective CCl_4_ treated group, *p* < 0.001.
