# Supplementary material for: Estimation of Bioactive Compound, Maslinic Acid by HPTLC, and Evaluation of Hepatoprotective Activity on Fruit Pulp of Ziziphus jujuba Mill. Cultivars in India
Source: Evid Based Complement Alternat Med. 2016 Jan 21;2016:4758734. doi: 10.1155/2016/4758734 (PMC4745332; doi:10.1155/2016/4758734)

TABLE S1. Validation data of HPTLC method for the estimation of maslinic acid.

| Intermediate precision (% RSD, n=6) | 0.82 |
| --- | --- |
| Calibration range (ng per spot) | 150-750 |
| Regression equation | Y= 29.616 + 0.343*X |
| Correlation coefficient | 0.9903 |
| Repeatability of Standards (% RSD , n = 6) | 0.97 |
| Repeatability of Samples (% RSD, n = 6) | 0.93 |
| Limit of Detection (LOD) (ng per spot) | 30 |
| Limit of Quantitation (LOQ) (ng per spot) | 87 |
| Robustness (% RSD, n = 3) | 0.89 |

TABLE S2: Study of intra-day and inter-day precision for maslinic acid

| Concentration (ng per band) | Intra-day (% *RSD*, n=6) | Inter-day (% *RSD*, n=6) |
| --- | --- | --- |
| 150 | 2.89 | 3.17 |
| 450 | 3.41 | 4.09 |
| 750 | 1.15 | 2.87 |

TABLE S3: Accuracy of the method for maslinic acid

| Sample Extract | Amount of maslinic acid (ng)^a^ | | | Recovery (%)^a^ | Average Recovery (%)^a^ |
| --- | --- | --- | --- | --- | --- |
|  | sample | Added | In mixture |  |  |
| *Umaran* | 640 ± 20.89 | 320 | 948 ± 11.28 | 98.75 | 97.41 |
|  | 640 ± 20.89 | 640 | 1260 ± 15.01 | 96.88 |  |
|  | 640 ± 20.89 | 960 | 1855 ± 13.45 | 96.61 |  |
| *Chhuhara* | 455 ± 25.66 | 227.5 | 623 ± 8.89 | 91.35 | 93.69 |
|  | 455 ± 25.66 | 455 | 870 ± 10.03 | 95.60 |  |
|  | 455 ± 25.66 | 682 | 1070 ± 12.21 | 94.11 |  |
| *Gola* | 670 ± 15.42 | 335 | 941 ± 10.74 | 96.62 | 94.37 |
|  | 670 ± 15.42 | 670 | 1263 ± 17.28 | 94.25 |  |
|  | 670 ± 15.42 | 1005 | 1545 ± 23.08 | 92.24 |  |
| *Sannur* | 647 ± 19.25 | 323.5 | 903 ± 21.89 | 93.09 | 94.61 |
|  | 647 ± 19.25 | 647 | 1263 ± 17.28 | 93.35 |  |
|  | 647 ± 19.25 | 970 .5 | 1575 ± 13.09 | 97.40 |  |
| *Mehrun* | 651 ± 27.01 | 325.5 | 913 ± 14.45 | 93.55 | 96.10 |
|  | 651 ± 27.01 | 651 | 1270 ± 11.56 | 97.54 |  |
|  | 651 ± 27.01 | 976.5 | 1582 ± 22.81 | 97.20 |  |
| Wild | 215 ± 25.43 | 107.5 | 297 ± 16.18 | 92.07 | 93.22 |
|  | 215 ± 25.43 | 215 | 397 ± 12.43 | 92.33 |  |
|  | 215 ± 25.43 | 322.5 | 512 ± 14.09 | 95.26 |  |
| *Kadaka* | 222 ± 17.34 | 111 | 312 ± 9.45 | 93.69 | 95.32 |
|  | 222 ± 17.34 | 222 | 428 ± 10.89 | 96.40 |  |
|  | 222 ± 17.34 | 333 | 532 ± 9.42 | 95.86 |  |
| *Apple* | 202 ± 17.05 | 101 | 282 ± 11.32 | 93.07 | 95.08 |
|  | 202 ± 17.05 | 202 | 386 ± 8.93 | 95.54 |  |
|  | 202 ± 17.05 | 303 | 488 ± 10.43 | 96.63 |  |

^a^ Mean ± SD (n=3).

FIGURE S1: HPTLC estimation of maslinic acid, A: Linear range of calibration plots for maslinic acid; B) Chromatograph of maslinic acid; C) Overlaid spectra of standard maslinic acid and sample of jujube.


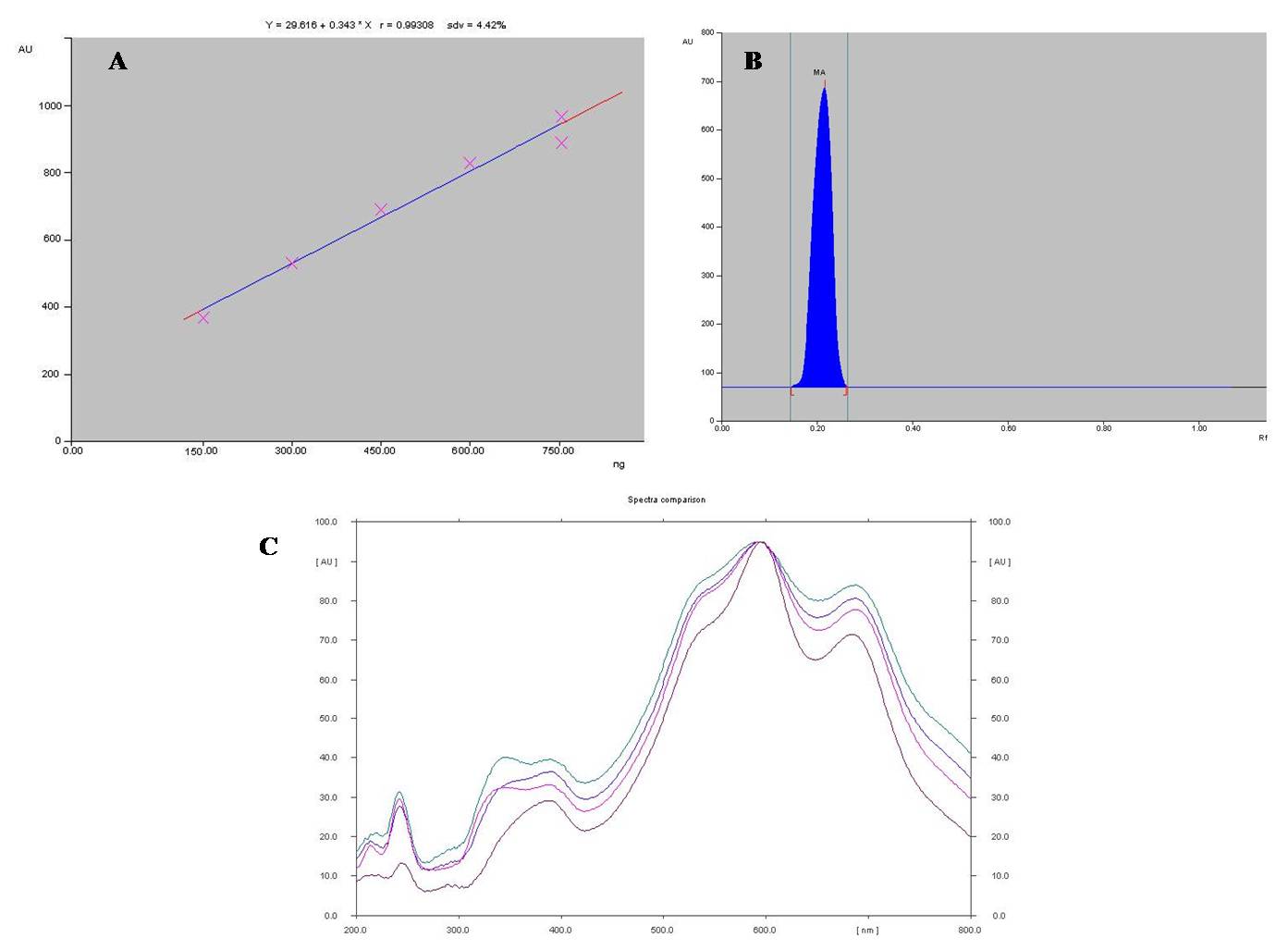

Supplement: Supplementary file 1 — HPTLC method was validated in terms of peak purity, precision, LOD, LOQ and accuracy according to ICH guidelines (2005). The method was specific for analysis of active principle maslinic acid in fruit pulp samples extract of eight cultivars of Z. jujuba. [file 4758734.f1.docx]
